# A Human(e) Factor in Clinical Decision Support Systems

**DOI:** 10.2196/11732

**Published:** 2019-03-19

**Authors:** Tim Bezemer, Mark CH de Groot, Enja Blasse, Maarten J ten Berg, Teus H Kappen, Annelien L Bredenoord, Wouter W van Solinge, Imo E Hoefer, Saskia Haitjema

**Affiliations:** 1 Laboratory of Clinical Chemistry and Haematology University Medical Center Utrecht Utrecht University Utrecht Netherlands; 2 Department of Anesthesiology University Medical Center Utrecht Utrecht University Utrecht Netherlands; 3 Department of Medical Humanities University Medical Center Utrecht Utrecht University Utrecht Netherlands

**Keywords:** clinical decision support, big data, artificial intelligence, machine learning, deep learning, precision medicine, expert systems, data science, health care providers

## Abstract

The overwhelming amount, production speed, multidimensionality, and potential value of data currently available—often simplified and referred to as *big data* —exceed the limits of understanding of the human brain. At the same time, developments in data analytics and computational power provide the opportunity to obtain new insights and transfer data-provided added value to clinical practice in real time. What is the role of the health care professional in collaboration with the data scientist in the changing landscape of modern care? We discuss how health care professionals should provide expert knowledge in each of the stages of clinical decision support design: data level, algorithm level, and decision support level. Including various ethical considerations, we advocate for health care professionals to responsibly initiate and guide interprofessional teams, including patients, and embrace novel analytic technologies to translate *big data* into patient benefit driven by human(e) values.

## Introduction

Although medical data collection and interpretation used to be the domain of health care professionals, the broad availability of health data in unprecedented amounts has significantly and irrevocably changed the landscape of modern care. Even patients now start to collect their own health data using, for instance, smart watches or apps, which may become an important source of health data in the future.

The craft of translating information into the right diagnosis and corresponding treatment is daily routine for health care professionals. It entails collecting the relevant data for each individual patient, integrating this information with pre-existing knowledge, drawing a conclusion, and initiating appropriate treatment in dialogue with the patient. A significant portion of medical training is dedicated to learning how to distinguish relevant from irrelevant information to ultimately make the best decision possible. Yet, the overwhelming amount, production speed, multidimensionality, and potential value of data currently available (often simplified and referred to as *big data*) exceed the limits of understanding of the human brain.

Conversely, developments in data analytics and computational power provide the opportunity to obtain new insights and transfer data-provided added value to clinical practice in real time. Such systems are called clinical decision support (CDS) and can broadly be defined as “information systems designed to aid in the clinical decision-making process, by integrating different sources of health information such as Electronic Health Records, laboratory test results, etc” [1]. CDS systems come in many forms and functions, but all share the aim of generating clinically relevant outcomes based on input data. A decision can be supported by a rule or a model as simple as an if-then rule (eg, built-in reference values for laboratory measurements) or a complex prediction model (eg, artificial intelligence [AI] pointing radiologists to possible incidental findings). The corresponding output of a CDS system varies from showing the generated prediction as input for a clinical decision (eg, automatically generated early warning scores) to acting upon the decision without human interference (eg, an implantable cardioverter defibrillator).

Recent reports on CDS systems in radiology and pathology are promising. Computers can, for example, support radiologists in interpreting mammograms or help pathologists in the classification of brain tumors [2,3]. Google recently also received the Food and Drug Administration’s approval for the introduction of a diabetic retinopathy algorithm based on retinal imaging [4]. Moreover, development of complex algorithms now starts to transcend beyond imaging specialties [5].

If the computer seems to know better anyhow, should we fully abolish medical curricula and focus on data scientists who develop CDS systems, with lay people gathering the information required for them, entitling the computer to do the interpretation instead? Probably not. There are at least two human beings present when a medical decision is made: a patient and a health care professional. Recently, the role of the patient as a disease *experience* expert [6] and his or her role in shared decision making have come into awareness. Here, we focus on the role of health care professionals and their expert knowledge. Throughout this paper, we will briefly touch upon various ethical issues. However, we strongly feel ethical considerations pertaining to algorithmic decision making deserve a discussion of their own, and kindly refer the reader to a recent overview on this topic [7]. In this paper, we show that a well-designed CDS system needs expert knowledge of health care professionals in all 3 phases of development: data, algorithm, and decision support (Table 1). Moreover, in the era of CDS, we advocate for health care professionals to responsibly initiate and guide interprofessional teams, including patients, and embrace novel analytic technologies to translate *big data* into patient benefit driven by human(e) values.

**Table 1:** This table shows the 3 levels in the building process of a clinical decision support system and some examples of where clinical expert knowledge of health care professionals plays a role in each of these levels.

Level and example of issue		Example of expert knowledge
**Data level**		
	Laboratory thresholds	Hemoglobin reference range to diagnose anemia
	Derived measurements^a^	Body mass index
	Diagnostic codes	Grouping of related diagnoses in a study population
	Jargon	Same abbreviations having different meanings
	Temporality	Glucose values are highly dependent on the time of day (eg, pre- or postprandial)
**Algorithm level**		
	Methodological choices	How to handle missing data (eg, missing not at random)
	Feature engineering^a^	Constructing relevant derived variables from raw data (eg, torsades de pointes, Wolff-Parkinson-White syndrome)
	Artifacts	For example, oxygen saturation of zero caused by a slipping pulse oximeter, switched leads in an electrocardiogram
**Decision support level**		
	Interpretation of model output	Risk probability of 0.75 requires a warning (*amber light*) in a CDS^b^ system
	Degree of autonomy	Tuning of implantable cardioverter defibrillator
	Knowledge on *usefulness*	Weighing a CDS system’s advice to treat while considering quality of life versus treatment burden in elderly cancer patients in a shared decision-making context

^a^Derived measurements may occur at the data level but also at the algorithm level; the former being undesirable because any manipulation at the data level may result in a loss of information.

^b^CDS: clinical decision support.

**Table 2:** Table comparing different types of clinical data on some points important to clinical decision support systems.

Clinical decision support issues	Electronic health record free-text/unstructured data (eg, clinical notes)	Registry/trial data (eg, case record forms case record forms and questionnaires)	Structured data/electronic health record (eg, lab values and smoking status)
Context completeness	Excellent: contextual information can be included.	Poor: context is essentially absent as a priori interpretation is an integral part of recording data in case record forms.	Depends on implementation. Context may be lost because of predetermined categorization.
Machine readability	Poor: information is mostly useful for case-specific usage by humans. May require text mining/text retrieval to convert to a machine-readable format.	Good: data are uniformly formatted and can be parsed by computers.	Excellent: data can be parsed or directly used by computers.
Translatability (between institutions)	Poor: free text contains jargon-specific, ambiguous abbreviations (eg, PCI: percutaneous coronary intervention/prophylactic cranial irradiation).	Excellent: trial data are usually collected using a standardized protocol, allowing for interoperability between institutions.	Good: lab values can be converted using reference values. Structured data, such as smoking and hypertensive status, can be reformatted for interoperability.
Noise resistance	Very poor: These type of data are very sensitive to *interobserver* noise (eg, personal abbreviations, spelling mistakes, and personal focus in recording certain types of information).	Excellent: data are recorded in a standardized way, designed to prevent noise.	Good: data are often machine-derived or recorded in a standardized way. However, bias because of differences in information-recording habits among health care professionals may arise.
Availability for reuse/general applicability	Excellent: these type of data are readily available, contain a lot of context (see Context completeness), and can thus be repurposed for a variety of applications.	Limited: trials are designed and conducted for one specific research question.	Excellent: these type of data are readily available and can thus be used for a plethora of purposes.
Design flexibility	Excellent: study design can be revisited if unanticipated bias effects arise. In this sense, bias could be *corrected* by altering the data selection.	Poor: study design is *hit-or-miss*. Bias cannot be *corrected* after the data recording process.	Excellent: study design can be revisited if unanticipated bias effects arise. In this sense, bias could be *corrected* by altering the data selection.

### Data

Developing a CDS system starts with data. Data come in many forms and sets (Table 2). Structured data such as numeric data (eg, laboratory measurements and blood pressure) or categorical data (eg, hypertension yes/no or educational level) are easiest to work with in a model. This is the first point at which expert knowledge of health care professionals may enter CDS development process. However, a substantial part of day-to-day clinical decisions is based on unstructured free-text entries, encompassing, for example, patient history and physical examination observations by doctors or regular notes from nurses. Although discouraged in modern electronic health record (EHR) systems, unstructured free-text clinical notes still provide irreplaceable information and context to health care professionals. Using free text introduces a number of challenges. Aside from the obvious ones, such as writing style and typos, medical text is incredibly site specific and can be highly biased. This phenomenon ranges from language- and country-specific abbreviations to jargon differences between 2 wards within the same hospital (eg, AF for atrial flutter and amniotic fluid or MS for mitral stenosis and multiple sclerosis). This is an understandable effect of rapid communication between health care professionals or of health care professionals taking personal notes to capture their train of thought. However, this leads to a given phrase, term, acronym, or abbreviation being context specific and having different meanings in different situations. Free-text interpretation, therefore, heavily depends on contextual expert knowledge.

### Data Sources

Widely used datasets for CDS systems include clinical trials and medical registries. Data collected within trials are of importance for a predefined research question. They are usually of high quality, may be stored in great detail, and are often richly annotated with expert knowledge (diagnostic codes and predetermined disease severity classifications). Medical registries are developed for quality control and research purposes. They are used to record a predefined limited number of variables for a specific group of patients, often focusing on particular conditions and diagnoses. Careful maintenance of research databases and registries allows for the collection of data from patients in a clean and systematic way according to protocol, preventing missingness and loss to follow-up as much as possible. However, because of their restrictive nature, research datasets and medical registries discard valuable contextual information, such as free-text notes, about included patients. Therefore, they show a limited, predefined scope of the patient’s condition. Furthermore, women and minorities are underrepresented in research datasets, and patients who are included can suffer from the Hawthorne effect (ie, altered behavior because of the fact that one is a study subject) [8]. The concept of research datasets and medical registries does not allow for flexibility in study design; the decision on what information to collect (and in what way) is single and final. Moreover, information beyond the scope cannot be added without considerable effort at a later moment (if anonymization or informed consent regulations do not prevent this at all).

Due to this rather *artificial* way of collecting data as compared with clinical care, research databases and registries are unsuited for the creation of broadly applicable CDS systems using increasingly complex models. Moreover, CDS systems preferably apply information that is already available to the care provider to aid in the clinical process without impeding it by requiring the collection of various additional data. Data from EHRs contain real-world data from clinical practice. EHR-based datasets are, therefore, more suitable for CDS system development. At the same time, EHR systems were designed as a virtual patient chart and not necessarily for reuse of the data they capture. As such, turning them into valuable EHR-based datasets takes careful and skilled data processing. For example, EHR data require more data cleaning (eg, how to handle *not at random* missing data—also a prime example of where clinical expert knowledge plays a vital role), careful assessment of informed presence bias (ie, acknowledgment of the bias introduced by the medical process), and decisions are to be made about how certain variables are derived from often unstructured data such as free text in EHR systems or clinical notes (eg, define diabetes mellitus and define hypertension) [9]. As only health care professionals themselves know about these inherent biases of working in an EHR, expert knowledge is indispensable. Table 2 compares different types of clinical data on a number of points important to CDS development. The Utrecht Patient Oriented Database in the University Medical Center Utrecht, the Netherlands, is an example of a routinely updated EHR-based database, containing data from multiple hospital sources of about 2.3 million patients (Multimedia Appendix 1, [10]). Utrecht Patient Oriented Database is curated by clinicians who use their expert knowledge in the design of the database to counter the known biases that are inherent to EHR data. Furthermore, they assist their clinical colleagues in transforming relevant data into meaningful variables to answer clinically relevant research questions and to develop CDS systems.

### Data Preprocessing

Before the data can be used to build a model, they need to be preprocessed. Preprocessing steps define variables from raw data that a model can use. During preprocessing steps, the expert knowledge of health care professionals is important to derive meaningful variables and values from the data. For example, disease activity variables need to be constructed because research guidelines and accompanying questionnaires are not regularly applied in clinical care. Furthermore, health care professionals may direct data scientists away from composite endpoints (eg, a patient has a 50% increased risk of pneumonia, pulmonary embolism, or chronic obstructive pulmonary disease) as they are less useful for CDS than specific endpoints that require specific actions (eg, a 50% increased risk of pneumococcal pneumonia). Moreover, the extraction of features from the data, such as differences in laboratory values over time, requires expert knowledge to determine appropriate time windows. Although the accuracy of algorithms generally increases if missing values and outliers are removed, the absence of data can carry value that only a health care professional is able to acknowledge, and the same holds true for outliers.

### Algorithm

After the selection of the right data to develop the CDS system, the next phase is to develop a model (ie, the *recipe* that describes the relationship between variables and outcome in the data) by using an algorithm (a predetermined computational method to derive such a *recipe* from the data). Depending on the complexity of the modeling task, model development usually contains a phase of model training and phase of model validation. In the training phase, a model that best fits the data (ie, makes the best predictions on the training data) is developed, and in the validation phase, tests are carried out to check whether the model is correct (ie, generalizes to the population). What constitutes a *good* prediction is dependent on the (clinical) research question (ie, identify all positive diagnoses at any cost or find a trade-off between cost and efficacy). It is common practice to test the model on a new dataset in the validation phase. This can be a previously unseen part of the total dataset or an entirely new dataset. Although modeling and algorithm development are not the natural habitat for most health care professionals, their knowledge and input are invaluable in this phase.

#### Simple Models

In simple models, the input of expert knowledge of health care professionals is well established. As mentioned before, the simplest form of decision models is *if-then* rules. Examples of such models include laboratory reference values based on statistical distributions of patient measurements (eg, if fasting glucose >11 mmol/L, then the patient probably has diabetes mellitus), medical risk scores (eg, if Glasgow Coma Scale is lower than 9, then consider intubating the patient), rule-based warnings for medication (eg, if the patient has impaired kidney function, then do not allow prescription for metformin), and alarms on the intensive care when vital sign thresholds are violated (eg, sound an alarm if saturation levels drop below 95%) [11]. When building these simple models into CDS systems, the thresholds and reference values need to be provided by health care professionals.

#### Complex Models

These traditional models and clinical scores are generally straightforward (Apgar score and Glasgow coma scale) to make them easily actionable, even in stressful situations. The beauty of their simplicity has ensured their broad application, but their sensitivity and specificity are unavoidably limited and usually include a substantial gray area. Moreover, most current models are based on regression or correlation measures that are less able to capture complex relationships in the data. The availability of machine learning offers novel approaches for developing medical models and risk scores. Machine learning refers to a group of statistical techniques that can be used to discern even complex patterns or regularities in data. They do so through an iterative process (in other words, the patterns are learned, hence machine *learning*) and produce a prediction model based on the learned patterns, which can then be incorporated in clinical support tools. [12,13]. In this complex type of modeling, input of expert knowledge from health care professionals may seem less obvious. However, model development is not a neutral process and even the values of health care professionals may be of additional benefit [7].

Machine learning algorithms can be roughly divided into 2 classes: (1) supervised learning algorithms that make use of prior (expert) knowledge about outcomes to guide the process and (2) unsupervised learning algorithms that aim to discover data patterns irrespective of model outcome.

Input data for supervised learning algorithms need to be labeled and selected manually (eg, positive/negative diagnosis, benign/malign, and concentration of inﬂammatory marker X) before modeling, and these data then constitute the outcome variable to predict for new cases. In other words, supervised learning systems rely heavily on expert knowledge [12,14]. *Supervision* is not only needed for the prelabeling of cases and noncases but also for statistical and methodological choices. Such choices include, for example, the choice of which algorithm to use and whether to normalize/standardize the data, and more algorithm-specific choices, such as the number of layers and nodes in a neural network or number of splits in a decision tree. Making appropriate choices on these aspects requires input from data scientists and medical scientists alike and will significantly affect the validity of the model. When the input variables in supervised models are selected by health care professionals and are based on prior knowledge and scientific evidence, supervised machine learning models may provide a safe ground for decision tools.

Unsupervised learning algorithms aim to uncover regularities in data without being guided by a prelabeling of the data (ie, clustering algorithms). The scope of this technique is often to discover novel subgroups within data and populations [12,14]. This approach is useful when information on the characteristics needed to discriminate between patients and controls is not yet available, or when one aims to find starting points for more fundamental scientific research. Therefore, this approach is usually used to find novel patterns in the data instead of making predictions and is thus generally more exploratory in nature. An advantage is, thus, that it allows for hypothesis-free or agnostic detection of patterns even when expert knowledge on the difference between subgroups is missing. Nevertheless, unsupervised systems can still profit from expert knowledge in the modeling process, as for example, clustering algorithms often require the user to preset the desired number of clusters, a decision that may be based on evidence of a known pattern in the population of interest.

A group of supervised and unsupervised techniques that is currently the state-of-the-art in machine learning is called deep learning. These techniques often involve artificial neural networks and attempt to learn increasingly *deep* representations of associations in the data. Deep neural networks (*deep nets*) are capable of automatically determining how to represent the input data in the best way for the question at hand. Theoretically, deep nets do not even require manual data preparation. In essence, the only requirements are to standardize numeric data and to encode categorical data into a numerical format interpretable by the algorithm. Deep learning is often used to recognize patterns in complex datasets that can subsequently be used by (supervised) machine learning models, for example, using clusters of a disease as outcome variables. In some experiments, deep learning methods have been shown to have superior prediction accuracy compared with other methods [15]. With EHRs as input, deep learning can improve prediction performance in modeling tasks that can be used for CDS [16]. Deep nets have, for example, already been proven useful in the computer-aided assessment and interpretation of medical images [4]. However, the rule of thumb *garbage in is garbage out* also applies when it comes to deep learning. If death is included in a model to predict readmission, it will probably come up as the most predictive variable, yet this might not be the actionable insight a health care professional is looking for. Guidance of health care professionals is needed when applying machine learning in the medical field.

#### Modeling Characteristics

Although all models remain specific for a given question (eg, what decision to support), building a CDS system is not a static process. It often includes rounds of major and minor changes of variables included and algorithmic fine-tuning. Moreover, some algorithms are never really finished. These algorithms are called *self-learning* and are designed to incorporate newly acquired data over time into their modeling processes. One of the reasons one may want to adapt an algorithm is spurious associations. Agnostic analyses that do not incorporate current understanding are prone to implement clinically irrelevant or even false associations with potentially deleterious consequences [17]. Outcomes of CDS models, therefore, need critical appraisal from experts regularly [17,18].

As health care professionals are responsible for the decisions they make, they highly value transparency of a model’s decision process and its development [19]. Whether or not the respective variables are shared with the health care professionals can be up for debate, as sharing of certain variables may lead to undesirable side effects. For example, an algorithm that states that a certain keyword in a patient history carries value as a warning, one may not want the health care professional to know this keyword to prevent it from being stated just to indicate a warning. A CDS system can be transparent to a greater or lesser extent. A CDS system that contains an algorithm that is too complex to comprehend can result in a so-called black box situation, where it is difficult or even impossible for a human brain to understand how the prediction model works. This renders validation of these black box algorithms extremely important. Unfortunately, there is a trade-off between attainable model complexity and model interpretability [20]. The opportunities that more complex models may provide should not be underestimated. To fully benefit from complex data and incorporate it into clinical practice, health care professionals may need to accept that the ultimate goal of thoroughly clinically validated predictive models in CDS systems may not be to be fully and completely interpretable or transparent but rather to be useful to a clinical purpose and influence patient outcomes. The process of model *development*, including choices that are made based on expert knowledge of both health care professional and data scientist, should always be as transparent as possible for all parties involved. Transparency of the development process may ultimately be critical for acceptance of CDS systems in clinical practice.

Moreover, depending on the complexity of the algorithm, internal (ie, on the same data) and external (ie, on other data) validation steps are vital before patients are exposed to the output. To what extent CDS systems need to be assessed as medical devices, according to their intended use, is still up for debate [21,22]. If-then CDS systems and CDS systems written to combine data into a visually attractive interface may be excluded from extensive clinical research but still need quality checks, regular revisiting of the algorithm, and piloting in clinical practice to ensure the right information is displayed for the right patient (does the algorithm take the most recent value from the table and did column names change). Furthermore, scientific evidence on validity and added value of the CDS system likely increases its use by health care professionals. Fortunately, such research is currently gaining traction in the medical community [23].

### Decision Support

Implementation and use of a CDS system consist of multiple steps, including presenting the algorithm output in a specific way, interpretation by the health care professional, and eventually, the medical decision that is made. A CDS system is not a bare model producing just an output (eg, *65%*). Almost always, it contains some level of interpretation. For example, laboratory measurements are often displayed in black, red, or blue to indicate whether they fall within or are higher/lower than a reference range. Risk percentages may be accompanied by a traffic light coloring scheme, indicating risk compared with a standard disease course. These manners of presentation (eg, how to report variables and what kind of user interface) are probably the most intuitive place to integrate the expert knowledge of a health care professional. Indeed, health care professionals and patients are often included in the user experience or user interface design phases to discuss implementation. However, this is frequently perceived too late in the CDS development process, and therefore, may yield an opposite effect.

Furthermore, model results have to be interpreted in a specific medical context before the CDS system can provide the actual tailored CDS and lead to action. This step is usually supervised by health care professionals. For instance, a cardiologist will double check the automated interpretation of the electrocardiogram (ECG) machine, and it is very likely that an eye specialist will supervise Google’s new diabetic retinopathy algorithm before any treatment is started [4]. Supervision of interpretation does not necessarily need a thorough understanding of the algorithm itself. Rather, it is the human intervention of integrating the contextual knowledge of the health care professional and, even more importantly, the patient’s wishes, before acting upon the algorithm’s output.

Most CDS systems do not (yet) act autonomously, so they need the attention of a health care professional to be effective. The highly technologically supported intensive care units provide ample evidence that getting the attention of a health care professional can be a challenge, as too many alarms can lead to alarm fatigue. Tweaking alert settings on an individual patient basis to make them meaningful as opposed to being perceived as a nuisance has been shown to improve compliance in critical care [11]. Incorporation of the multitude of emerging CDS systems into clinical practice needs streamlining and thorough knowledge of the medical process. Health care professionals should take initiative to lead interprofessional teams, deciding how and when to report CDS.

Unfortunately, systematic scientific evidence outlining what requirements a useful CDS system should meet is missing [24] and has been replaced by more or less anecdotic or empirical recommendations for many years. The *Ten Commandments of Clinical Decision Support* [25] lists factors as speed, anticipation of information need, integration into the workflow, or general ease-of-use type of advice in alerts. Moreover, negative advice, for example, an advice not to perform or order a particular test, is rarely accepted when no alternative is suggested, and the method of alert presentation has been found to be crucial to alert compliance [26].

Although such experience-based recommendations remain useful, the most important evidence for the usefulness of a CDS system that will influence acceptation by health care professionals will be its ability to influence clinical outcomes. Unfortunately, the evidence for CDS systems’ frequently purported advantages over *old-fashioned* clinical decision in improving clinical outcome, workload, and economic cost is scarce. However, CDS systems have been shown to improve health care processes and are the best way to decrease unnecessary clinical testing [27].

Acceptation of CDS by health care professionals depends on the degree to which they feel autonomous in their decision making. Rather than choosing colors for a user interface, being part of the development process, identifying the appropriate data, discussing model design, and validation may help health care professionals to feel in control in the midst of forces that are transforming daily clinical practice. A supportive organization with inspiring leadership encouraging involvement of health care professionals in the development of CDS systems stimulates this transformation.

## Discussion

With the rise of machine learning, and especially deep learning in CDS systems, it is perhaps tempting to let IT and data experts build CDS systems, redirecting health care professionals to merely gathering data. However, we have shown that human(e) health care professionals are still of paramount importance, as all phases of development and use of a CDS system requires the extensive expert knowledge of health care professionals. Health care professionals should not just be involved in implementing the CDS system into clinical practice but should be part of an interprofessional CDS development team from the start, initiating and guiding development through clinical demand and expert knowledge. They bring in the clinical decision they want the CDS system to support and help to understand the context in which variables are collected during routine care. Their interpretation is vital in extracting relevant variables from raw data and in avoiding the implementation of spurious associations in CDS systems. Moreover, as health care professionals want the best for their patients, they may even develop a sense of moral obligation to embrace strategies that unravel data complexities beyond their comprehension, as relying on methods that do not use the data to their full potential leads to potentially unused value for their patients. At the same time, both data scientists and health care professionals should be alert to cognitive biases provided by pre-existing expert knowledge. Indeed, interprofessional CDS development teams should be as inclusive as possible, as the values and preferences of the people involved influence the underlying model [28].

The hallmark of biology, variability, is complex to capture in a static algorithm, and a medical decision is not based on objective single data points but on subjective, context-sensitive longitudinal observations made by health care professionals during patient contact. This has consequences for the acceptance of autonomous CDS systems. Single measurements are not likely to lead to an acceptable *autonomous* action by a CDS system except for when doing nothing is likely to cause more harm (eg, an automated defibrillator that decides whether electric cardioversion is indicated based on ECG input and applies the appropriate therapy itself). Other accepted autonomous CDS systems, such as pacemakers or insulin pumps, gather continuous data, and thereby, learn and improve their efficacy for the individual patient. As longitudinal data collected by sensors inside and outside the hospital are becoming more ubiquitous, the value and applicability of accepted autonomous CDS systems enriched with these data are likely to improve. Still, the decision to implement a pacemaker and monitoring and tweaking its action and settings during follow-up are a doctor’s job, including contextual expert knowledge and the patient’s own preferences into the CDS system. Therefore, clinical reasoning is unlikely to ever be completely replaced by fully automated decision making through machine learning without human intervention. Even when expert knowledge is already embedded in medical data, and when the CDS system is clinically validated, the need for health care professionals to navigate the intricacies of incorporating expert knowledge in the ultimate clinical decision must not be underestimated. This includes implicit knowledge or *gut feeling* for which computability is limited [29]. Moreover, the final decision (how) to use the CDS is up to the health care professional and their patient.

Therefore, most CDS systems do not aim to replace health care professionals but are designed to support them. For example, the recent algorithms in the field of medical imaging preprocess data and take over tedious and simple tasks so that radiologists and pathologists can focus on more complex cases, acting more creatively. Given the recent developments in data protection regulations, health care professionals cannot easily be replaced by AI. The European General Data Protection Regulations state “The data subject shall have the right not to be subject to a decision based solely on automated processing, including profiling, which produces legal effects concerning him or her or similarly significantly affects him or her.” This regulation appears tailored to the medical profession. Health care professionals (but basically everybody processing data) have a responsibility to guard their patients against irresponsible implementation of data-driven technologies. This especially holds true for self-learning algorithms that self-adapt to the patient population without human intervention, which may autonomously change considerably over time (so-called *algorithmic drift*). What is the meaning of human intervention in this sense, if the health care professional has no insight at all into the opaque model? In this light, how can health care professionals still justify their actions? How does this translate to accountability?

Importantly, the decision of how to respond to a CDS system is a moral one, and moral considerations regarding when to treat or not to treat are the expertise of human beings rather than that of AI systems. A CDS system cannot decide whether *primum non nocere* applies to a specific situation as harm and good and quality of life depend on personal judgement, context, and preferences of human beings. Some patients may be willing to take a risk that others would not, including application of a CDS system with a black box algorithm to their specific case. This way, cultural difference may indicate the need for locally tweaked systems. People, whether patients or their loved ones, should participate in shared decision making, tailoring the usage and outcomes of CDS systems to their wishes. What is *best* for the patient depends on more than just the output of a CDS system.

In conclusion, it is of paramount importance that health care professionals initiate and guide the development and implementation of CDS in clinical care, as opposed to waiting to be overwhelmed by current technological advancements. Most data scientists are not medical experts, and vice versa. Therefore, data scientists and health care professionals should team up in an interprofessional fashion, preferably also including patients. Data scientists who enthusiastically welcome recent innovations in AI pose a bold claim and carry the burden of proof to equip health care with suitable CDS tools. Once health care professionals can be convinced of the added benefit of CDS for their patients, they may acknowledge the necessity and value of data collection, interpretation, and curation, so they may embrace their expanding role and further evolve from doctor knows best to doctor does best.

## Appendix

Multimedia Appendix 1 This table shows a number of indicative characteristics of the Utrecht Patient Oriented Database (UPOD) as of February 2018.

## Abbreviations

AI: artificial intelligence
CDS: clinical decision support
ECG: electrocardiogram
EHR: electronic health record
