# Supplementary material for: A Human(e) Factor in Clinical Decision Support Systems
Source: J Med Internet Res. 2019 Mar 19;21(3):e11732. doi: 10.2196/11732 (PMC6444220; doi:10.2196/11732)
Supplement: Multimedia Appendix 1 [file jmir_v21i3e11732_app1.pdf]

Supplemental Material

Supplemental table 1 *This table shows a number of indicative characteristics of the Utrecht Patient Oriented Database (UPOD) as of February 2018.*

UPOD Database Entity	Total
Patients (unique)	2.300.000
Clinical Patients (unique)	600.000
Hospitalizations	1.500.000
Lab orders	12.000.000
Lab tests	110.000.000
Medication orders (clinical)	6.000.000
Medication orders (policlinical)	4.500.000
Blood Cell Counts	2.400.000
Measurements	75.000.000
Procedures treatments	97.000.000
Diagnose Treatment Combinations (DBC, in Dutch healthcare)	3.500.000
